# Mass-Controlled Direct Synthesis of Graphene-like Carbon Nitride Nanosheets with Exceptional High Visible Light Activity. Less is Better

**DOI:** 10.1038/srep14643

**Published:** 2015-09-28

**Authors:** Zaiwang Zhao, Yanjuan Sun, Qian Luo, Fan Dong, Hui Li, Wing-Kei Ho

**Affiliations:** 1 Chongqing Key Laboratory of Catalysis and Functional Organic Molecules, College of Environmental and Biological Engineering, Chongqing Technology and Business University, Chongqing, 400067, China; 2 Engineering Research Center for Waste Oil Recovery Technology and Equipment, Ministry of Education, Chongqing Technology and Business University, Chongqing, 400067, China; 3 Department of Science and Environmental Studies, The Centre for Education in Environmental Sustainability, The Hong Kong Institute of Education, 10 Lo Ping Road, Tai Po, New Territories, Hong Kong, China

## Abstract

In the present work, it is very surprising to find that the precursors mass, a long overlooked factor for synthesis of 2D g-C_3_N_4_, exerts unexpected impact on g-C_3_N_4_ fabrication. The nanoarchitecture and photocatalytic capability of g-C_3_N_4_ can be well-tailored only by altering the precursors mass. As thiourea mass decreases, thin g-C_3_N_4_ nanosheets with higher surface area, elevated conduction band position and enhanced photocatalytic capability was triumphantly achieved. The optimized 2D g-C_3_N_4_ (CN-2T) exhibited exceptional high photocatalytic performance with a NO removal ratio of 48.3%, superior to that of BiOBr (21.3%), (BiO)_2_CO_3_ (18.6%) and Au/(BiO)_2_CO_3_ (33.8%). The excellent activity of CN-2T can be ascribed to the co-contribution of enlarged surface areas, strengthened electron-hole separation efficiency, enhanced electrons reduction capability and prolonged charge carriers lifetime. The DMPO ESR-spin trapping and hole trapping results demonstrate that the superoxide radicals (•O_2_^−^) and photogenerated holes are the main reactive species, while hydroxyl radicals (•OH) play a minor role in photocatalysis reaction. By monitoring the reaction intermediate and active species, the reaction mechanism for photocatalytic oxidation of NO by g-C_3_N_4_ was proposed. This strategy is novel and facile, which could stimulate numerous attentions in development of high-performance g-C_3_N_4_ based functional nanomaterials.

The confinement of electron transfer in two dimensional (2D) systems with unique properties has stimulated tremendous attention, particularly nanosheets and layered nanojunctions with thickness on the scale of several atoms^1^,^2^,^3^,^4^. An eminent incarnation of such atomically thin materials is graphene, and it has attracted great attention since the finding of freestanding graphene and the follow-up experimental confirmation that its charge carriers are indeed massless Dirac fermions^5^,^6^. Hitherto, the explorations of graphene-analogue 2D crystals have experienced an explosion of interest because much added-value may be brought by the translation of 2D atomic crystals into semiconductors^7^.

Polymeric carbon nitride, as layer materials, made up from infinite 1D chains (a zigzag-type geometry) of NH-bridged melem (C_6_N_7_(NH_2_)_3_) monomers was initially reported by Berzelius and termed “melon” by Liebig^8^,^9^. It is of great interest that graphite-like carbon nitride (g-C_3_N_4_), a prototypical 2D polymer featuring a semiconductor band gap of 2.7 eV, is in favour of visible light absorption, charge carriers generation, separation and transfer on the interface^10^,^11^,^12^,^13^. Moreover, nanosheets achieved by the delamination of 2D layered compounds have been regarded as a novel class of nanostructured materials owing to their unique structural characteristic of ultimate two-dimensional anisotropy with extremely small thickness in nanometer scale^14^. This feature often brings nanosheets with unique physicochemical properties due to the quantum confinement effect. For instance, it possesses exceptional mechanical, electronic, thermal, optical properties compared to bulk nanomaterials^15^. In photocatalytic reactions, nanosheets are particularly beneficial for enhancing photocatalysis efficiency. The merits of high specific surface for 2D marerials are beneficial for providing adequate reactive sites and shortening bulk diffusion distance for reducing the recombination probability of photo-driven electrons-holes pairs^16^. Because of the quantum confinement effect, the enlarged bandgap can enhance redox ability of charge carriers, which is a pivotal factor for enhance the photocatalytic activity. In addition, the lifetime of charge carriers will be prolonged, and the photophysical behavior of photo-excited charge carriers will be transformed, which is induced by the 2D structure and enlarged band gap^17^.

On one band, the nano/microstructure of g-C_3_N_4_ can be internally engineered by optimization of pyrolysis conditions (temperature, time, and atmosphere) and selecting specific precursors^18^. On the other hand, many external strategies have been developed, including templating approaches^19^, protonation with HCl^20^,^21^, doping with metal/nonmetal elements^22^,^23^, dye sensitizing^24^, copolymerization^25^,^26^, hybridization^27^,^28^,^29^,^30^,^31^,^32^ and etc. However, template methods usually suffer from tedious processes, including template modification, precursor attachment, and template removal, which may lead to a cumbersome process and over-expenditure. Moreover, the external methods that could enhance the g-C_3_N_4_ must be under additive assistances, which make the fabrication process complicated.

Hence, it is indispensable to develop a facile and environmental benign approach for g-C_3_N_4_ fabrication with highly enhanced photocatalysis. Most of the factors influencing the microstructure and photocatalytic activity of g-C_3_N_4_ have been systematically investigated, such as the types of precursor, pyrolysis condition, exfoliation, doping and other modification strategies. However, the crucial factor of precursor mass has been long overlooked.

Unexpectedly, we discovered that the precursor mass exerted a crucial role in governing the microstructure and enhancing the photocatalytic performance of g-C_3_N_4_. We explored the unique effects of thiourea mass on the morphology and electronic structure of g-C_3_N_4_ in detail. The resultant sample were investigated in terms of micro-morphology, electronic structure, photophysical properties and photocatalytic capability. It is amazing to find that high quality g-C_3_N_4_ nanosheets can be easily obtained just by diminishing the precursor amount. The as-prepared g-C_3_N_4_ nanosheets possess enhanced band gap, promoted charge separation efficiency and prolonged charge carriers life time, which results in exceptionally high photocatalytic capability. The excellent photocatalytic capability also outperforms that of the bulk g-C_3_N_4_, the well-known BiOX, (BiO)_2_CO_3_ and noble metal modified (BiO)_2_CO_3_. It is obvious that less precursor brings more. This favorable factor can be significant guidance for advance g-C_3_N_4_ material. As the method facile and environmental benign, it is of great potential for accelerating the pace for g-C_3_N_4_ practical application in environmental and energetic applications.

## Results and Discussion

### Structure and morphology

**Figure 1:**
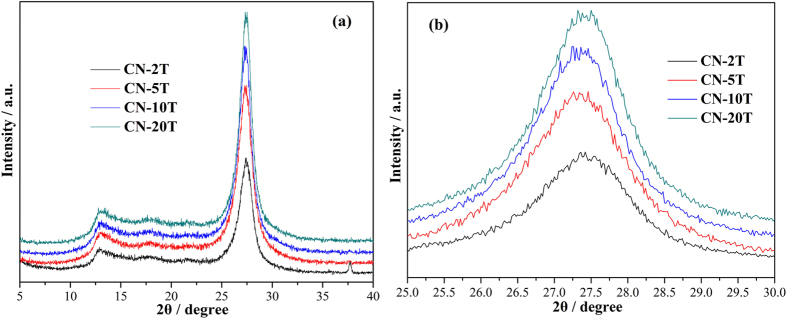
XRD pattern of g-C_3_N_4_ obtained from different masses of thiourea (**a**) and enlarged view of (002) peak (**b**).

Figure 1a reflects the XRD patterns of the as-prepared g-C_3_N_4_ samples treated under different precursor masses (2, 5, 10 and 20 g). All of the g-C_3_N_4_ samples in Fig. 1a possess similar diffraction patterns, suggesting that all samples are similar in crystal structure^33^. The strongest (002) peak around 27.5° is observed, which typically indicates the graphite-like stacking of the conjugated aromatic units of CN^33^. A typical (100) diffraction peak around 13.0° can be indexed to (100) peak of graphitic materials, in accordance of the in-plane tri-s-triazine units which formed one-dimension (1D) melon strands^34^,^35^. Further observation on an enlarged view of (002) peak (Fig. 1b) demonstrates that the diffraction angle 2θ of (002) peak gradually increases, which can be ascribed to the more complete condensation of thiourea when the precursor mass was decreased. The typical (002) peak is 27.20° for CN-20T, 27.28° for CN-10T, 27.35° for CN-5T and 27.42° for CN-2T respectively, indicating the interplanar distance tends to decrease. The reduced interplanar distance of CN-2T will shorten the charge transport distance from the bulk to the surface, beneficial for the final products separated from the photocatalysts, which is highly desirable for photocatalytic applications^36^,^37^.

**Figure 2:**
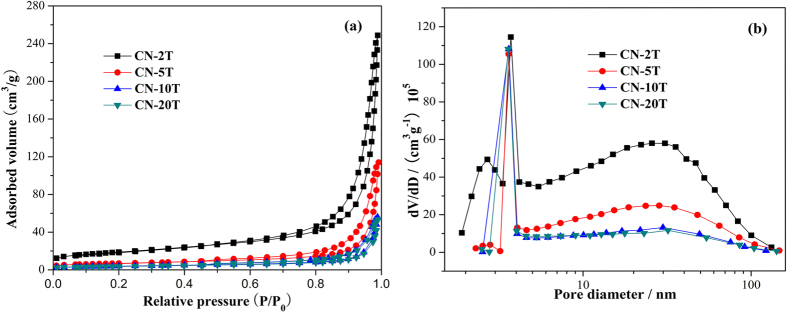
N_2_ adsorption-desorption isotherms of CN-2T, CN-5T, CN-10T and CN-20T (**a**) and the corresponding pore-size distribution curves (**b**).

**Figure 3:**
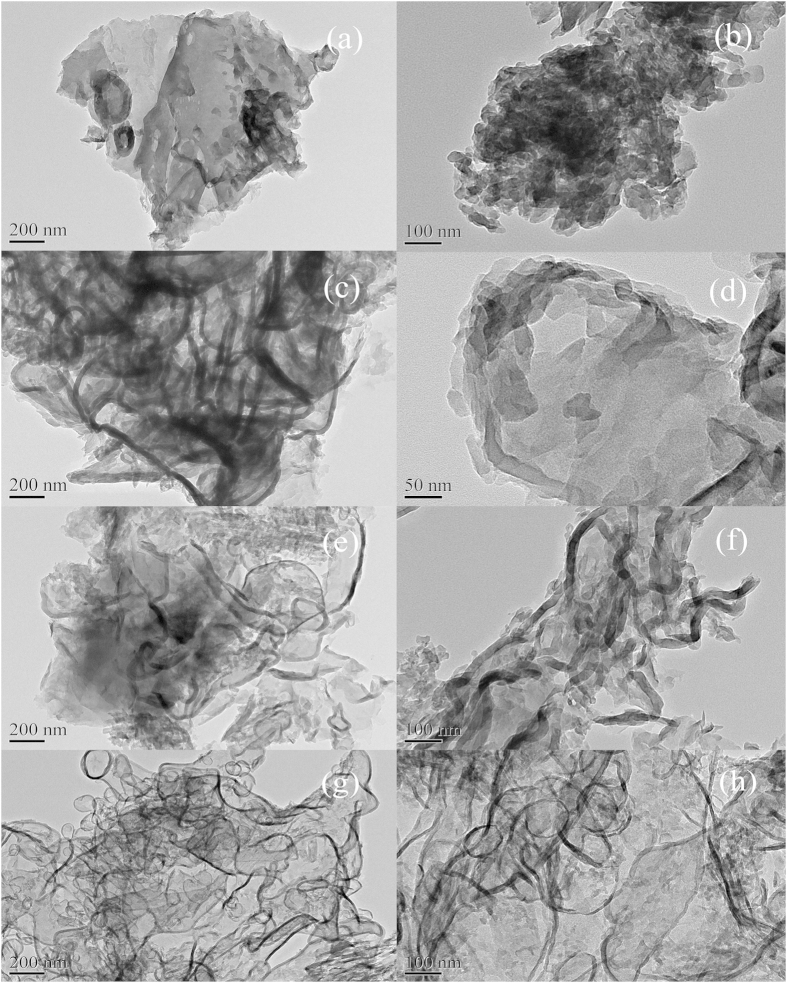
TEM and magnifying view of pattern of g-C_3_N_4_ obtained from different masses of thiourea, (**a,b**) for CN-20T, (**c,d**) for CN-10T, (**e,f**) for CN-5T and (**g,h**) for CN-2T.

Figure 2 depicted that the nitrogen adsorption-desorption isotherms and Barrett-Joyner-Halenda (BJH) pore-size distribution of as-prepared samples. All samples exhibit nanoporous architecture, as reflected in Fig. 2a,b. The nitrogen adsorption-desorption isotherms (Fig. 2a) for all samples are assigned to type IV (Brunauer, Deming, Deming, and Teller, BDDT classification) suggesting the presence of mesopores (2–50 nm)^37^,^38^. As the thiourea mass decreased, the hysteresis loops shift to the region of lower relative pressure accompanied by the enhanced hysteresis loops areas, indicating the formation of enlarged mesopores architectures^37^. The hysteresis loops assigned to type H_3_ indicate the formation of slit-shaped pores from the aggregates of plate-like particles. Notably, the apparent enlargement of surface areas and mesopores volumes can be observed when diminished the precursors mass (Fig. 2b and Table 1). These results are well in agreement with the sheet-like morphology of g-C_3_N_4_ presented in SEM (Fig. 3). Besides, the correlations among surface area, pore volume, and precursor mass of g-C_3_N_4_ samples are summarized in Table 1. The S_BET_ was increased from 12 m^2^/g (CN-20T) to 66 m^2^/g (CN-2T), as well as the pore volume enlarged from 0.083 cm^3^/g (CN-20T) to and 0.39 cm^3^/g (CN-2T), when the precursor mass was diminished from 20 to 2 g. In addition, transformations of peak pore-size distribution (PSD) (Fig. 2b) of g-C_3_N_4_ corresponding to diverse precursor masses further confirm the introduction of mesopores architecture. The peak intensity of mesopores with diameters about 30.0 nm gradually increased as the precursor mass was decreased. Especially, the PSD curve of CN-2T is quite broad (from 2 to 100 nm) with small mesopores (~2.7 and ~3.7 nm) and large mesopores (~30.8 nm). The small mesopores can be assigned to the porosity within the nanoscale sheets (Fig. 3a), and the large mesopores assigning to the porosity between packed layers. Less is better. The high surface area and pore volume will be beneficial for augmenting the number of active sites and accelerating the transfer of the intermediates and final products, which could exhibit higher photocatalytic capability^16^.

The typical TEM images of the as-prepared samples are shown in Fig. 3. It demonstrates that all as-synthesized samples obtained from diverse precursors are composed of irregularly curved layers. The nanosheets become thinner and the architecture turn fluffier, as the precursor mass gradually decreased. Figure 3a reflects that the CN-20T sample is composed of tightly overlapped bulk layers, further demonstrated by the magnifying view (Fig. 3b). When the precursor mass is decreased to 10 g, the structure of CN-10T turns fluffy and the nanosheets becomes thinner, producing nori-like structure (Fig. 3c,d). Besides, as the precursor mass is decreased to 5 g, the thickness of the nanosheets will further be reduced, and large amount of smooth nanosheets overlap irregularly together forming tremella-like layers (Fig. 3e,f). Unexpectedly, when the masses of the thiourea are further reduced to 2 g, thickness of CN-2T nanosheets was nearly reduced to several nanometers scale, resulting in extremely fluffy and transparent architecture (Fig. 3g,h).

Thus, a conclusion can be drawn that in a semi-closed system, less precursor mass contributes to g-C_3_N_4_ with smaller 2D nanosheets, unique porous architecture and higher specific surface area. However, no solid was remained in crucible as thiourea mass was less than 2 g. On the basis of observation, we proposed a reaction mechanism to clearly elucidate the reaction processes during the pyrolysis of thiourea in a semi-closed system. In converting thiourea into g-C_3_N_4_, the synergistic effects of pyrolysis-generated self-supporting atmosphere^36^ and air molecules (mainly nitrogen and oxygen) exert great significance in tailoring the microstructure and properties of g-C_3_N_4_. In semi-closed system, the retained nitrogen and oxygen molecule can be well-distributed in the pyrolysis-generated self-supporting atmosphere^39^. Fewer precursors can produce less self-supporting atmosphere. Therefore more sufficient nitrogen and oxygen concentration in the reaction system can generate an array of gas bubbles, splitting when it grows up, in resulting in stripping g-C_3_N_4_ layers into smaller layers, as well as forming more fluffy structure (Fig. 3)^37^. The thickness of g-C_3_N_4_ samples will be remarkably reduced by the layer-by-layer exfoliation process^12^ in semi-closed system with less precursor masses. Moreover, the fewer precursors in semi-closed system, the more space can be used for self-supporting atmosphere growing and diffusion, which can be also a contribution to fluffy structure of g-C_3_N_4_.

**Figure 4:**
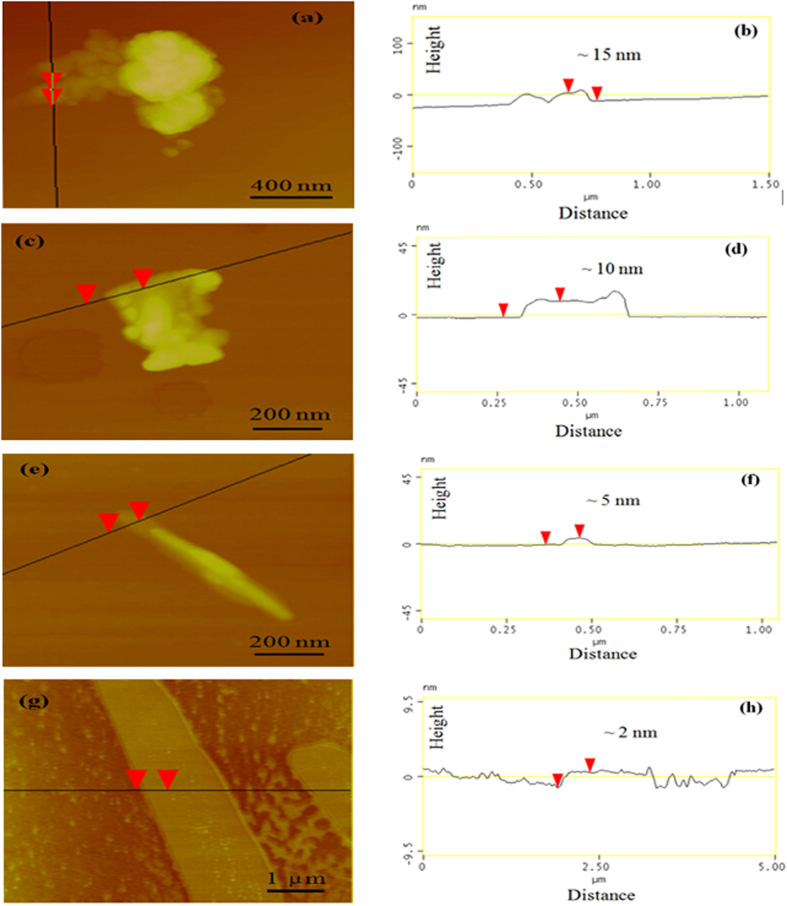
The typical AFM image and the corresponding thickness analysis results of g-C_3_N_4_ nanosheets obtained for different mass of precursors, (**a,b**) for CN-20T, (**c,d**) for CN-10T, (**e,f**) for CN-5T and (**g,h**) for CN-2T.

However, for less than 2 g thiourea, the scarce pyrolysis-generated self-supporting atmosphere will be further diluted to lower concentration. These dilute pyrolysis-generated gases quickly escaped the system without further reaction, and the products would be decomposed at high temperature, leading to no solids residue. The typical AFM images and thickness analysis (Fig. 4) reflect that the nanosheets thickness of g-C_3_N_4_ is apparently decreased for about 15 nm for CN-20T, 10 nm for CN-10T, 5 nm for CN-5T to 2 nm for CN-2T. This result further demonstrates that the precursor amount is a pivotal factor in controlling the thickness of g-C_3_N_4_. Decreasing the precursors mass is favorable for generation of thinner g-C_3_N_4_ nanosheets.

### Chemical composition

Figure S1 in the Supporting Information shows the FT-IR spectra of CN-2T, CN-5T, CN-10T and CN-20T. We can observe that the weak absorption at 700–800 cm^−1^ region are assigned to the bending vibration mode of CN heterocycles, and the characteristic out of plane bending vibration mode of the triazine units at 810 cm^−1^ are found for all the samples^37^. The absorption bands in the range of 1200–1600 cm^−1^ are assigned to stretching mode of C-N heterocycles, and the broad bands in the range of the 3000–3700 cm^−1^ region are attributed to the adsorbed H_2_O molecules and N-H vibration^39^. For fewer samples, the polymerization reaction of thiourea can be more complete resulting in stronger vibration of the C_6_N_7_ units, consistent with XRD results. In addition, absorption bands assigned to sulfur bond (such as -SH, -SN, -SC) have not been detected, indicating that the sulfur element in thiourea of CN-20T, CN-10T, CN-5T and CN-2T is completely released during thermal treatment. From XPS spectra, the final ratio between C and N for CN-20T, CN-10T, CN-5T, CN-2T ranges from 0.746 to 0.752, close to the theoretical ratio of C and N in g-C_3_N_4_, which indicates that the as-prepared samples have high purity.

**Figure 5:**
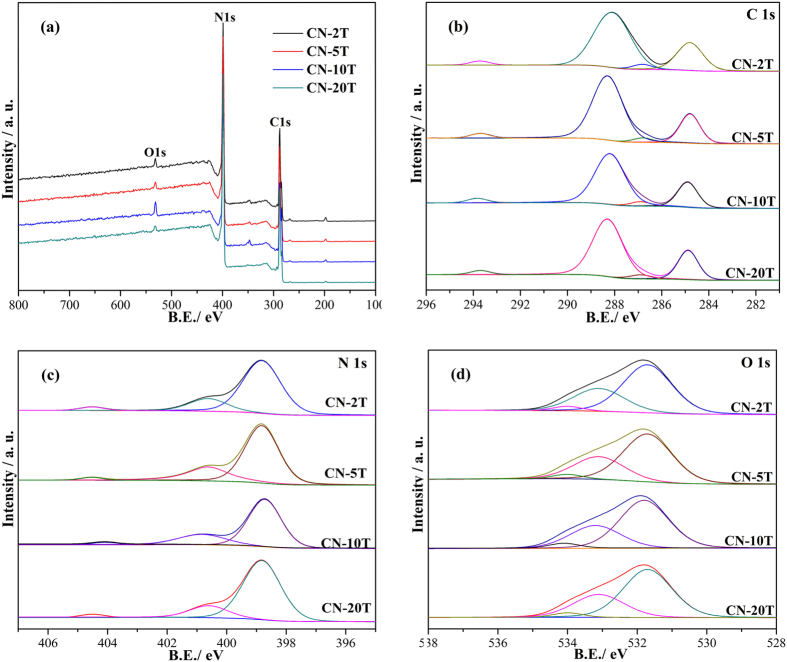
XPS spectra of CN-2T, CN-5T, CN-10T and CN-20T samples, survey (**a**), C1s (**b**), N1s (**c**), O1s (**d**).

The XPS measurements were carried out to determine the chemical state of the elements for all samples. Figure 5a represents the full survey spectra of C, N and O elements for CN-2T, CN-5T, CN-10T, and CN-20T. Peaks assigned to S species cannot be observed for each sample, further indicating that the sulfur in thiourea was completely released during heating treatment. The C1s spectra (Fig. 5b) clearly reflect the similar four peaks for all the obtained samples. The peak located at 284.8 eV relates to adventitious carbon species, and the other three peaks at 286.8, 288.3 and 293.7 eV, corresponding to (C)_3_-N, C–N–C and N–C–O coordination in the g-C_3_N_4_ lattice, respectively^40^. The N1s region (Fig. 5c) can be fitted into four peaks, which can be ascribed to C−N−C (398.8 eV), tertiary nitrogen N-(C)_3_ (400.6 eV), and π-excitations (404.5 eV), respectively^41^. As shown in Fig. 5d, the O1s spectra of all g-C_3_N_4_ samples can be clearly divided into three peaks with binding energies of 531.7, 533.1 and 534 eV. The major peak at 533.1 eV is assigned to C–N–O, assign to partial oxidation of the low polymerized CN during thermal exfoliation in air^40^. The other two peaks at 531.7 and 534 eV are assigned to surface -OH groups and adsorbed H_2_O, which is consistent with FT-IR observation^40^.

### Optical properties and band structure

**Figure 6:**
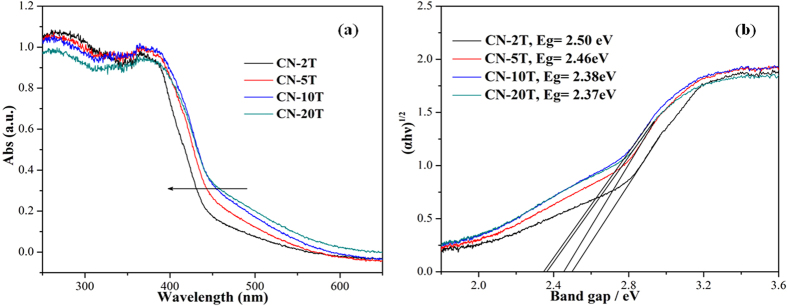
UV−Vis DRS (**a**) and plots of (αhν)^1/2^ versus photon energy (**b**) of CN-2T, CN-5T, CN-10T and CN-20T.

The optical properties of CN-20T, CN-10T, CN-5T, CN-2T samples were investigated by UV−vis DRS (Fig. 6a). As demonstrated in Fig. 6a, the optical absorption spectra shows that all samples feature an intrinsic semiconductor absorption in the blue region of the visible spectra, corresponding to band gap transitions from valence band to conduction band. The absorption edges of g-C_3_N_4_ samples change with the variation of precursor masses. Figure 6b demonstrates the band gap energy, estimated from the intercept of the tangents to the plots of (αhν)^1/2^ vs photon energy. The band gap energy of the samples increase from 2.37 eV for CN-20T, 2.38 for CN-10T, 2.46 for CN-5T, to 2.50 for CN-2T depicted in Fig. 6a,b. The hypsochromic shift of the absorption edges reflects the quantum confinement effects of the thermally induced thinner nanosheet structure as is evidenced in TEM (Fig. 3) and AFM (Fig. 4), which is assigned that the size and thickness of g-C_3_N_4_ are in nanoscale region^42^. The enlargement in band gap could subsequently boost the photocatalytic redox ability.

**Figure 7:**
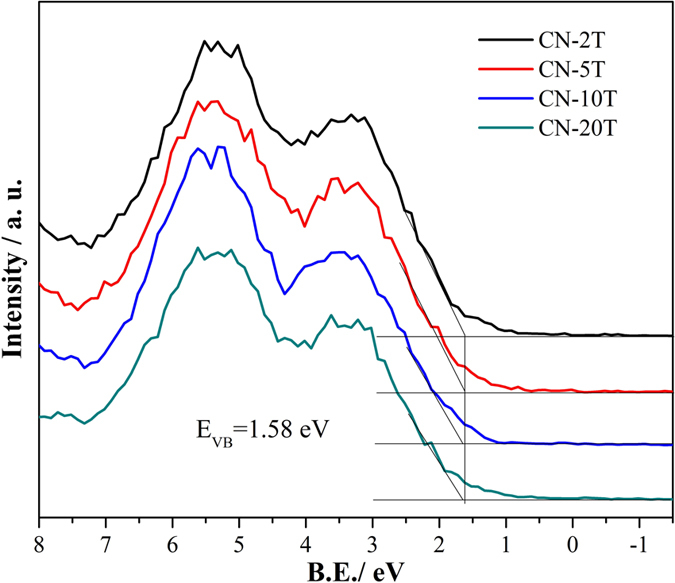
VB XPS (b) of CN-2T, CN-5T, CN-10T and CN-20T.

To investigate the electronic structures and reduction ability of the electrons, we measure VB XPS (Fig. 7) of the as-synthesized samples. The VB XPS reflects that the valence band maximums (VBM) of all samples are the same (1.58 eV), which demonstrates that photo-oxidation abilities of the holes for all samples are equal.

**Figure 8:**
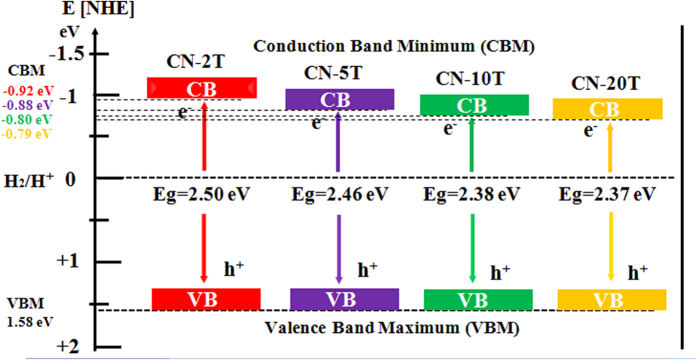
Illustration of the band gap structures of CN-2T, CN-5T, CN-10T and CN-20T.

Figure 8 illustrates the valence band maximum (VBM) and conduction band minimum (CBM) potentials of CN-2T, CN-5T, CN-10T and CN-20T, as the band structures perform a pivotal role in the photocatalytic performance, as well-known. Generally speaking, the more positive the value of VB, the higher the mobility of holes produced, along with the better the photo-oxidation efficacy of holes. As showed in Fig. 8, the VBM potentials of all samples are the same, demonstrating that photo-oxidation abilities of the holes for all samples are equal. Nevertheless, the CBM potentials shift to more negative values. The CBM potentials values are decreased from −0.79, −0.80, −0.88 to −0.92 eV, which is associated with the degree of polymerization of π-conjugated polymeric network in fewer precursors. In general, the more negative the value of CB, the stronger reductive power of the photo-excited electrons, contributing to strengthen the photo-driven electron-holes separation efficiency due to charge carrier good transport ability^43^.

**Figure 9:**
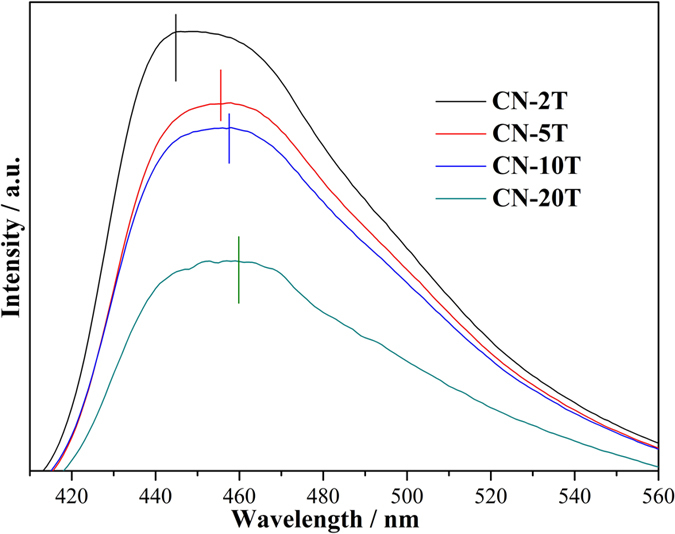
Room temperature PL spectra (Excitation light source:280 nm) of CN-2T, CN-5T, CN-10T and CN-20T.

PL spectra have been widely applied to investigate the charge carrier trapping, migration, and transfer of electron-hole pairs in semiconductors. PL emission results from the recombination of electron-hole pairs. Figure 9 depicts the room temperature PL spectra of g-C_3_N_4_ obtained from different mass of precursors. Emission peaks centered at around 440–455 nm for all samples originate from direct band-to-band transitions. It is remarkable that CN-2T sample exhibits the highest PL emission peak, which is associated with its less structural imperfection on account of more complete condensation of thiourea, as evidenced by XRD and FT-IR. Note that more complete condensation of thiourea minimized the number of structural defects (e.g., uncondensed **−**NH_2_, **−**NH groups), which could capture the electrons or holes, hence resulting in high radiative PL emission. Figure 9 also reflects that the PL peak position located at 463, 455, 445 and 442 nm for CN-20T, CN-10T, CN-5T and CN-2T, respectively. This hypsochromic-shift of emission peak is consistent with the enhancement of band gap energy of these samples (Fig. 6b), which is ascribed to quantum confinement effect^44^.

**Figure 10:**
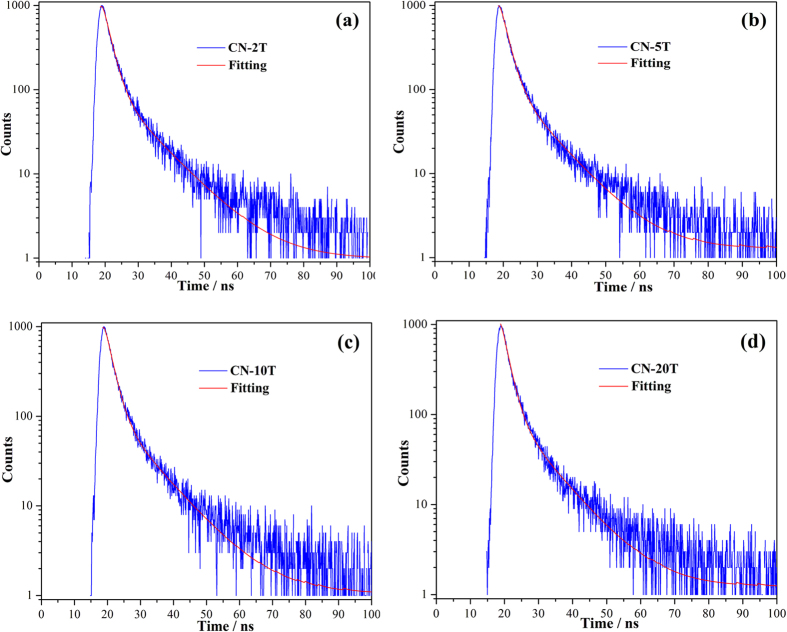
(**a**–**d**) Ns-level time-resolved PL spectra monitored at 450 nm under 420 nm excitation at 77 K for (**a**) CN-2T, (**b**) CN-5T, (**c**) CN-10T, and (**d**) CN-20T.

To understand the photophysical characteristics of photo-excited charge carriers, the ns-level time-resolved fluorescence decay spectra of CN-2T, CN-5T, CN-10T, and CN-20T were recorded, as shown in Fig. 10(a–d). By fitting the decay spectra, the radiative lifetimes with different percentages can be determined (Table 2). As the precursors masses decrease, the short lifetime of the samples is prolonged from 1.8 ns for CN-20T, 1.9 ns for CN-10T and CN-5T to 2.0 ns for CN-2T. Moreover, the long lifetime of charge carriers is increased from 9.5 ns for CN-20T to 10.4 ns for CN-2T. The prolonged radiative lifetime of the charge carriers is really crucial in improving the probability of their involvement in photocatalytic reaction before recombination. The increased lifetime of charge carriers is associated with the more negative conduct band potentials and enhanced charge transfer induced by CN-2T. This prolonged lifetime would afford to improve the probability of electrons or holes captured by reactive substrates to initiate the photocatalytic reactions.

### Photocatalytic activity, stability and mechanism

**Figure 11:**
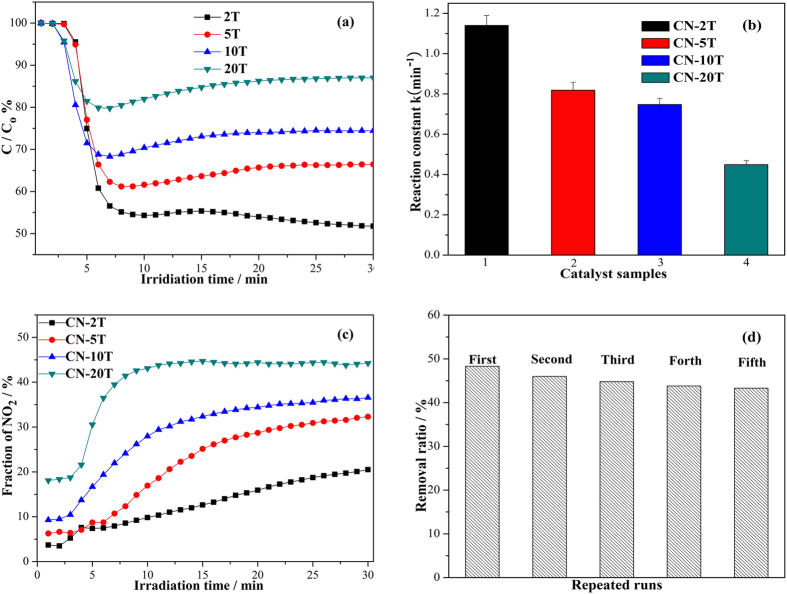
Photocatalytic activities (**a**) and apparent rate constants (**b**) of CN-2T, CN-5T, CN-10T and CN-20T samples for NO degradation in air under visible light illumination (NO concentration: 600 ppb); (**c**) Monitoring of the fraction of NO_2_ intermediate over g-C_3_N_4_ samples during photocatalytic reaction and (**d**) stability test of the CN-2T under 5 cycles irradiation with visible light (λ > 420 nm).

The photocatalytic activity of the as-prepared g-C_3_N_4_ samples was evaluated by removing gaseous NO (concentration: 600 ppb) under visible-light irradiation in a continuous reactor for air purification. Figure 11a depicts the variation of NO concentration (C/C_0_%) with irradiation time over g-C_3_N_4_ samples treated form different precursor masses. Previous investigation indicated that NO could not be degraded without photocatalyst under light irradiation or with photocatalyst for lack of light irradiation^33^,^45^,^46^. When the g-C_3_N_4_ is irradiated in the absence of NO gas, no products can be detected, which indicates that the products (NO_2_, NO_3_^−^) are originated from the photocatalysis of g-C_3_N_4_. In the presence of photocatalyst, the photo-generated reactive radicals react with NO, converting NO to the final product of HNO_3_^40^. Figure 11a reveals that the all g-C_3_N_4_ samples of CN-2T, CN-5T, CN-10T and CN-20T demonstrate visible light photocatalytic activity toward NO removal, in accordance with the facts that g-C_3_N_4_ has a suitable band gap that can be directly excited by visible light. Figure 11a also shows that the NO concentration for all samples decreased rapidly in 5 min. The slight decrease in activity during 5 to 15 min can be ascribed the gradual generation of reaction intermediates that may occupy the active sites of photocatalysts. When the reaction reached equilibrium, the photocatalytic activity was kept almost unchanged. Moreover, the NO removal ratio of g-C_3_N_4_ samples regularly increases from 12.7, 25.5, 33.6 to 48.3% for CN-20T, CN-10T, CN-5T and CN-2T after 30 min irradiation as a equilibrium. The optimized activity of CN-2T samples sharply outperforms that of BiOBr (21.3%)^47^, as well as the (BiO)_2_CO_3_ (18.6%) and Au/(BiO)_2_CO_3_ (33.8%)^48^, demonstrating that diminishing the amount of precursor is an effective strategy to enhance the photocatalytic activity of g-C_3_N_4_.

To better understand the reaction kinetics of the NO degradation catalyzed by g-C_3_N_4_ photocatalysts, the experimental data were fitted by a first-order model, according to the true that the value of the rate constant *k*_app_ commonly gives an indication of the capability of the nanocomposites photocatalyst. Figure 11b gives the values of the rate constants *k*_app_ of all g-C_3_N_4_ samples. CN-2T reflects the highest apparent *k*_app_ of 1.13 min^−1^, which was about 2.7 times as that of CN-20T sample (0.42 min^−1^).

The reaction intermediate of NO_2_ is monitored online during photocatalytic oxidation of NO presented in Fig. 11c. Photocatalytic oxidation of NO to NO_2_ is not beneficial for application as NO_2_ is more toxic. To promote real application, NO conversion to NO_2_ should be inhibited. The NO should be oxidized to final product of NO_3_^−^. As we can see in Fig. 11c, 42.2% of NO is conversed to NO_2_ for CN-20T sample and the selectivity of NO to final NO_3_^−^ is 57.8%. This is not so good for application. However, the conversion ratio of NO to NO_2_ is decreased to 20.0% for CN-2T and the selectivity of NO to final NO_3_^−^ is increased to 80.0%, which is favorable for application. This enhanced NO conversion ratio over CN-2T can be ascribed to the more negative conduction band of the graphene-like g-C_3_N_4_ nanosheets. The electrons on conduction band of CN-2T could induce the generation of more reactive species (•O_2_^−^) (evidenced below by ESR spectra) because of the more negative conduction band minimum, thus advancing the oxidation of intermediate NO_2_ to final NO_3_^− ^49. The final oxidation products (nitric acid or nitrate ions) can be simply washed away by water wash. Note that the NO concentration in the outlet was decreased gradually when the photocatalytic reaction was going on, due to the continual conversion of NO to NO^3−^. What is more, the NO concentration would reach minima till the photocatalytic reaction reached equilibrium. The slight rising of NO concentration was due to the accumulation of NO^3−^ product on the catalyst surface, occupying the active sites^45^,^49^,^50^. After long-term irradiation, the NO concentration in the outlet would reach a steady state. The stability of the optimized photocatalyst was tested in an extended experiment (Fig. 11d). The high capability was reproducible, and the material showed excellent stability during 5 times cycle testing. In addition, we also measured XRD spectra of the CN-2T samples after stability testing, and the result reflected that the architecture of the optimized samples is firmly stable (Fig. S2).

**Figure 12:**
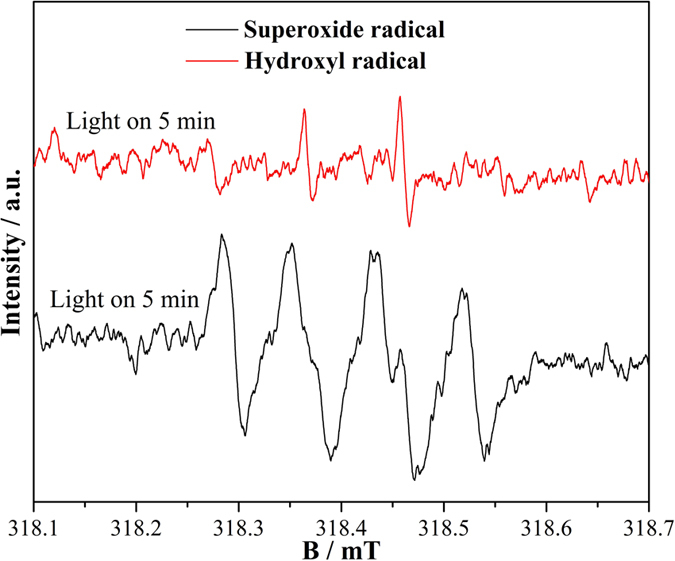
DMPO spin-trapping ESR spectra of CN-2T in methanol dispersion for •O_2_^−^ detection and in aqueous dispersion for •OH detection under visible light illumination.

To elucidate the main reactive species responsible for the NO removal reaction, ESR technique was employed for CN-2T in reaction systems. Figure 12 shows ESR spectra measured as the effect of light irradiation on CN-2T photocatalyst at room temperature in air. DMPO (5,5-dimethyl-1-pyrroline N-oxide) is nitrone spin trap generally used for trapping radicals due to the generation of stable free radicals, DMPO-•OH or DMPO-•O_2_^− ^46. Under visible light illumination, four strong characteristic peaks with similar intensity of DMPO-•O_2_^−^ adduct can be detected, which testifies that the massive production of -•O_2_^−^ produced via the reduction of O_2_ with photo-generated electrons (Eqs. (1) and (2))^51^. Besides, the DMPO-•OH adduct signals with intensity ration of 1:2:2:1 were clearly observed, but the intensity of DMPO-•OH is significantly weaker than that of DMPO-•O_2_^−^. Based on the fact that the potential energy of valence band (VB) holes (1.58 eV) from g-C_3_N_4_ is lower that of OH^−^/•OH (1.99 eV) and H_2_O/•OH (2.37 eV), the holes cannot directly oxidize OH^−^ or H_2_O into •OH. Alternatively, the •OH should be generated via the transformation of •O_2_^−^, shown in Eqs (3) and (4). Hence it can be deduced that •OH radicals detected in the water system come from the reactions between •O^2−^ and H_2_O. The •O_2_^−^ species is the main reactive radical, while •OH species exert a subordinate role in photocatalytic oxidation of NO (Eqs. (1, 2, 3, 4)), reflected by the signal of the origination and strength of the two radicals. Moreover, judging from the electrode potential (Eφ), the VB holes of g-C_3_N_4_ sample might also oxidize NO because the Eφ_VB_ (about 1.58 V vs. NHE) of g-C_3_N_4_ is more positive than Eφ (NO_2_/NO, 1.03 V vs. NHE), Eφ (HNO_2_/NO, 0.99 V vs. NHE), and Eφ (HNO_3_/NO, 0.94 V vs. NHE)^51^. The holes as active species are further confirmed by trapping experiment as shown in Fig. S3. When the typical hole trapping agent (KI, 1 wt.%) is mixed with the CN-2T sample, the photocatalytic activity is decreased rapidly, indicating photo-generated holes are one of the active species for g-C_3_N_4_ photocatalysis. Based on aforementioned discussion, the reaction mechanism of photocatalytic oxidation of NO by g-C_3_N_4_ is proposed as presented in Eqs. (1, 2, 3, 4, 5, 6, 7, 8).
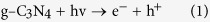

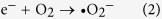

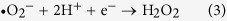

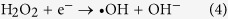

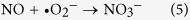

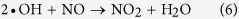

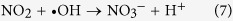

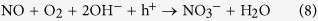


Above all, the exceptional high photocatalytic activities of CN-2T can be explained as the co-contribution of the 2D nanosheets-like architectures, reduced interplanar distance, higher surface area, unique porous architecture, elevated CBM, enhanced electron-holes separation efficiency, as well as prolonged lifetime of charge carriers. Firstly, for the g-C_3_N_4_ sample treated at smaller mass of thiourea, the nanosheet-like structure (Fig. 3a) and reduced interplanar distance (Fig. 1b) can facilitate the photo-induced electrons and holes transportation, thus lowering the recombination rates of the photo-driving hole-electron pairs^16^. Secondly, the nanosheets with high surface area (Fig. 3) can advance the pollutant adsorption^52^ and provide more active sites for intermediates diffusion^53^. Thirdly, numerous large mesopores provides more spaces for quick diffusion of reactants and intermediates^54^. Pivotally, the promotion of the conduct band minimum could largely expedite the reduction capability of electrons contributing in more •O_2_^−^ species generation, as well as enhancing the photo-driving hole-electron separation and prolonging the lifetime of charge carriers^12^. All these favorable factors co-contribute to the exceptional high photocatalytic performances of CN-2T.

Beyond expectation, by facilely tailoring thiourea mass, we have successfully engineered the nanostructures of g-C_3_N_4_ with exceptional high visible light photocatalytic performance. This strategy not only provides a facile method for optimizing g-C_3_N_4_ with superior photocatalytic capacity, but also a guidance for fabricating multi-functional materials in areas such as solar energy conversion, photosynthesis, and catalyst support. We have done initial experiments on other g-C_3_N_4_ precursors, such as dicyandiamide and urea. For these precursors, the results indicate that less amount of precursor is also beneficial for enhancement of photocatalytic activity of g-C_3_N_4_, which implies that the present method is general for synthesis of graphene-like g-C_3_N_4_.

## Conclusion

The precursor mass, a critical factor governing the micro-architecture of g-C_3_N_4_, has long been overlooked. Considering the unique effects of precursor mass, we developed a novel strategy for direct production of graphitic carbon nitride, with excellent photocatalytic capability for NO purification under visible-light illumination. This unique effects of precursor mass on the microstructure and photocatalytic activity of g-C_3_N_4_ were firstly revealed. It was surprising to find that the morphology and the photocatalytic activity of g-C_3_N_4_ were highly dependent on the precursor masses. A new concept for nanomaterials synthesis was proposed: Less is better, and meanwhile this founding can offer a guidance for obtaining g-C_3_N_4_ with excellent performance. When diminishing thiourea mass from 20 to 2 g, the visible light photocatalytic capability of 2D g-C_3_N_4_ nanosheets toward gaseous NO purification was conspicuously enhanced. The exceptional high visible-light activity of CN-2T can be ascribed to the contribution of accelerated electrons transportations, enhanced electron-hole separation efficiency, strengthened electrons reduction capability and prolonged charge carriers lifetime. This strategy is novel, environmental benign, easily-available without the aid of co-factors, which will stimulate extensive attentions in environmental and energetic domain.

## Methods

### Synthesis of g-C_3_N_4_

All reagents used in this study were analytical grade. In a typical synthesis, thiourea powder of different masses (2, 5, 10 and 20 g) were put into four different alumina crucibles (50 ml in volume) with a cover, respectively, and then heated to 550 °C at a heating rate of 15 °C/min in a muffle furnace for 2 h. After the reaction, the alumina crucible was cooled to room temperature. The resultant g-C_3_N_4_ were collected and ground into fine powders. The g-C_3_N_4_ samples prepared from different precursor masses (2, 5, 10 and 20 g) were labelled as CN-2T, CN-5T, CN-10T and CN-20T, respectively.

### Characterization

The crystal structures were analyzed by X-ray diffraction with Cu-Kα radiation (XRD: model D/max RA, Japan). The morphological structure was analyzed by transmission electron microscopy (TEM: JEM-2010, Japan). The UV-vis diffuse reflection spectra were measured through a Scan UV-vis spectrophotometer (UV-vis DRS: UV-2450, shimadzu, Japan) with an integrating sphere assembly. The 100% BaSO_4_ was chose as reflectance sample. The atomic force microscopy (AFM) study in the present work was performed by means of MultiMo de-V (Veeco Metrology, Inc.). FT-IR spectra were measured with a Nicolet Nexus spectrometer embedded in KBr pellets. The Fluorescence spectrophotometer (FS-2500, Japan) was used to investigate the photoluminescence spectra taking Xe lamp as the excitation source with optical filters. The surface properties and the total density of the state (DOS) distribution of the valence band were recorded by the X-ray photoelectron spectroscopy with 150 W Al-Kα X-ray radiation (XPS: Thermo ESCALAB 250, USA). The shift of the binding energy was calibrated using an internal standard of C1s level of 284.8 eV. The specific surface areas were measured through the nitrogen adsorption-desorption on a nitrogen adsorption apparatus (ASAP 2020, USA). The samples were degassed at 150 °C before measurement. The FLsp920 Fluorescence spectrometer (Edinburgh Instruments) was utilized to obtain time-resolved photoluminescence spectra with the excitation at 420 nm. The ESR measurement (FLsp920, England) was recorded by mixing g-C_3_N_4_ in a 50 mM DMPO (5, 5’-dimethyl-1-pirrolin e-N-oxide) solution with aqueous dispersion for detection of DMPO-•OH and methanol dispersion for detection of DMPO-•O_2_^−^.

### Visible light photocatalytic DeNOx activity

The photocatalytic capability of ppb-level NO purification was investigated in a continuous flow reactor. The rectangular reactor with a volume capacity of 4.5 L (30 cm × 15 cm × 10 cm) was manufactured with stainless steel and covered with Saint-Glass. A 150 W commercial tungsten halogen lamp was vertically placed above the reactor. The UV beam was cut off by adopting a UV cut-off filter (420 nm). A certain amount of photocatalyst (0.2 g) was coated onto two dishes (12.0 cm in diameter). Then the coated dish was placed at 70 °C in a drying oven to remove water of the suspension. NO gas was supplied by a compressed gas cylinder at a concentration of 100 ppm (N_2_ balance). The NO concentration of 600 ppb was acquired by the air stream dilution. The desired relative humidity (RH) level (50%) of the NO flow was controlled by passing the zero air streams through a humidification chamber. The gas streams were pre-mixed completely through a gas blender. Mass flow controllers were utilized to control the flow rate at 2.4 L/min. When the adsorption-desorption equilibrium reached, the lamp was turned on. The concentration of NO was continuously measured by a chemiluminescence NOx analyzer (Thermo Environmental Instruments Inc., 42i-TL). The removal ratio (*η*) of NO was determined with *η* (%) = (1 − C/C_0_) × 100% (1), where C and C_0_ are the concentrations of NO in the outlet steam and the feeding stream, respectively. The photocatalytic kinetics of NO purification is a pseudo-first-order reaction at low NO concentration as ln(C_0_/C) = *k*_app_*t*, where *k*_app_ stands for the apparent rate constant.

## Additional Information

**How to cite this article**: Zhao, Z. *et al.* Mass-Controlled Direct Synthesis of Graphene-like Carbon Nitride Nanosheets with Exceptional High Visible Light Activity. Less is Better. *Sci. Rep.* **5**, 14643; doi: 10.1038/srep14643 (2015).

## Supplementary Material

Supplementary Information

## Figures and Tables

**Table 1:** The *S*_BET_, pore volume, peak diameter, band gap value, NO removal ratio and NO_2_ fraction of g-C_3_N_4_ samples as well as that of the references.

Sample name	*S*_BET_(m^2^/g)	Pore volume(cm^3^/g)	Peak diameter(nm)	Band gap(eV)	NO *η* (%)	NO_2_ fraction(%)
CN-2	66	0.39	2.7/3.7/30.8	2.50	48.3	20.5
CN-5	24	0.18	3.6/28.4	2.46	33.6	32.3
CN-10	13	0.087	3.6/30.0	2.38	25.5	36.6
CN-20	12	0.083	3.6/32.0	2.37	12.7	44.2
BiOI^47^	9	0.029	–	1.77	2.5	–
BiOBr^47^	11	0.023	–	2.76	21.3	–
(BiO)_2_CO_3_^48^	46	0.113	–	–	18.6	–
Au/(BiO)_2_CO_3_^48^	42	0.114	–	–	33.8	–

The data for BiOI, BiOBr, (BiO)_2_CO_3_ and Au/(BiO)_2_CO_3_ were collected from related references.

**Table 2:** Kinetics of emission decay parameters of CN-2T, CN-5T, CN-10T and CN-20T.

Samples	Component	Life time (ns)	RelativePercentage (%)	χ^2^
CN-2T	τ_1_	2.0	72.5	1.059
	τ_2_	10.4	27.5	
CN-5T	τ_1_	1.9	74.0	1.052
	τ_2_	10.1	26.0	
CN-10T	τ_1_	1.9	71.5	1.044
	τ_2_	9.5	28.5	
CN-20T	τ_1_	1.8	70.8	1.024
	τ_2_	9.5	29.2	
