# Supplementary material for: Mass-Controlled Direct Synthesis of Graphene-like Carbon Nitride Nanosheets with Exceptional High Visible Light Activity. Less is Better
Source: Sci Rep. 2015 Sep 28;5:14643. doi: 10.1038/srep14643 (PMC4585959; doi:10.1038/srep14643)
Supplement: Supplementary Information [file srep14643-s1.doc]

**SuppLEMENTARY Information**

Mass-Controlled Direct Synthesis of Graphene-like Carbon Nitride Nanosheets with Exceptional High Visible Light Activity. Less is Better.

Zaiwang Zhao ^1^, Yanjuan Sun ^1^, Qian Luo ^1^, Fan Dong^1,2^*, Hui Li^†^ & Wing-Kei Ho ^3^

^1^ Chongqing Key Laboratory of Catalysis and Functional Organic Molecules, College of Environmental and Biological Engineering, Chongqing Technology and Business University, Chongqing, 400067, China.

^2^ Engineering Research Center for Waste Oil Recovery Technology and Equipment, Ministry of Education, Chongqing Technology and Business University, Chongqing, 400067, China.

^3^ Department of Science and Environmental Studies, The Centre for Education in Environmental Sustainability, The Hong Kong Institute of Education, 10 Lo Ping Road, Tai Po, New Territories, Hong Kong, China.

*To whom correspondence should be addressed. Phone: +86 23 62769785 605. Fax: +86 23 62769785 605. E-mail: [dfctbu@126.com](mailto:dfctbu@126.com)


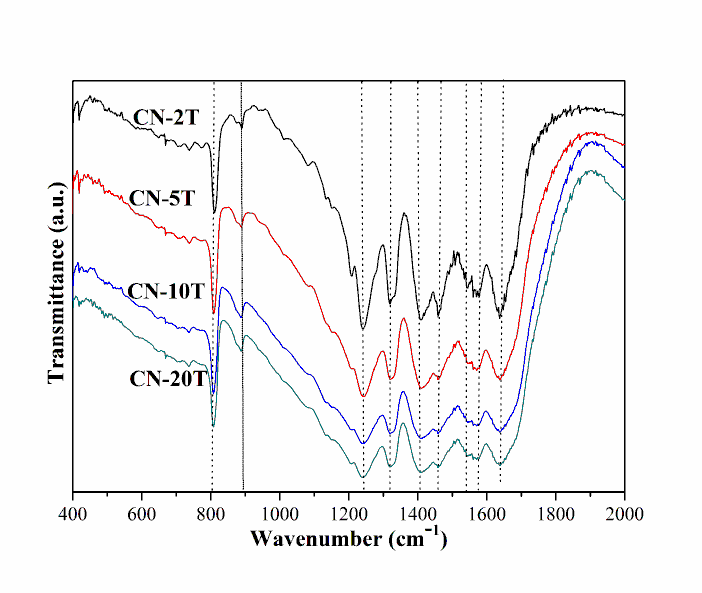

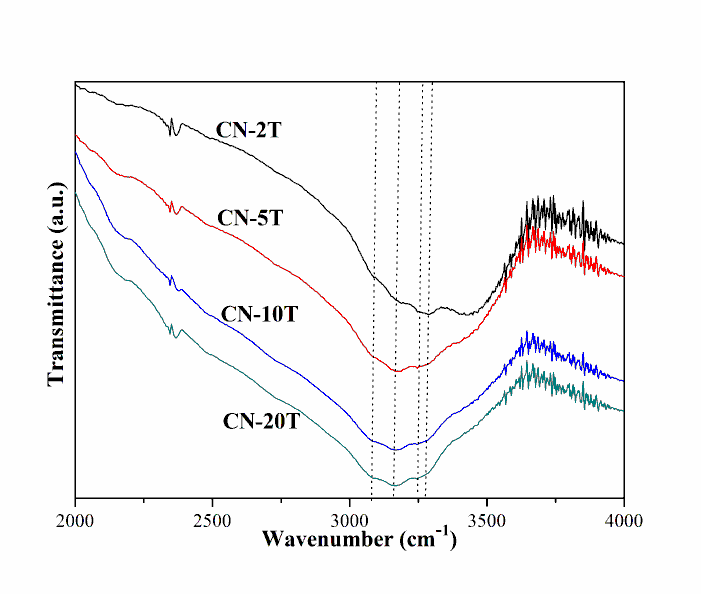


**(a)**

**(b)**

**Figure. S1** FT-IR spectra of g-C_3_N_4_ samples treated from different masses of thiourea in the range of 400-2000 cm^-1^ (a) and 2000-4000 cm^-1^ (b).


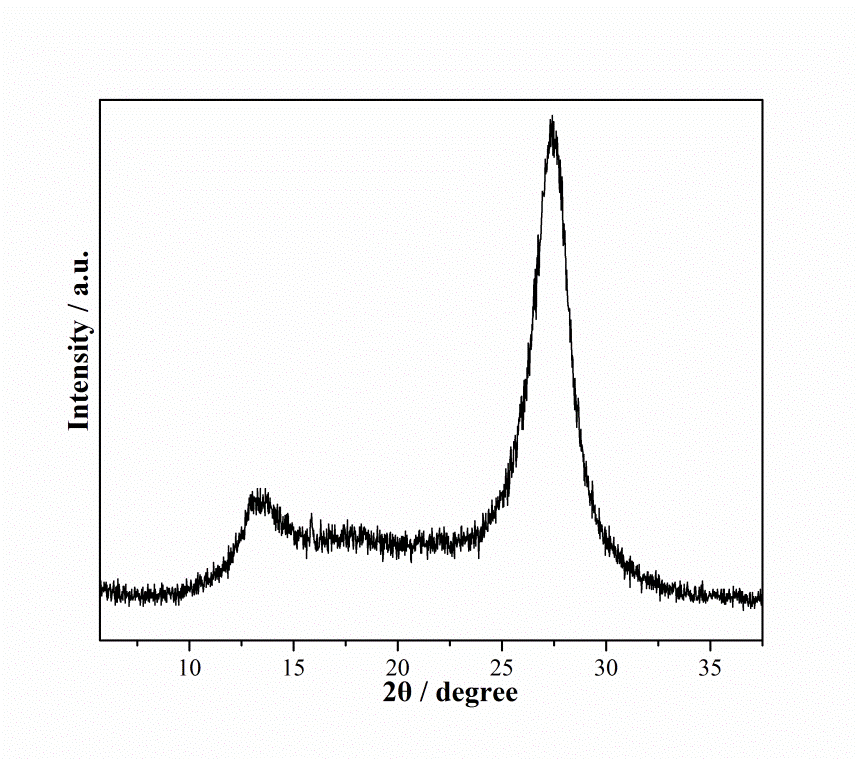


**Figure. S2** XRD spectra of the optimized CN-2T samples after stability testing.


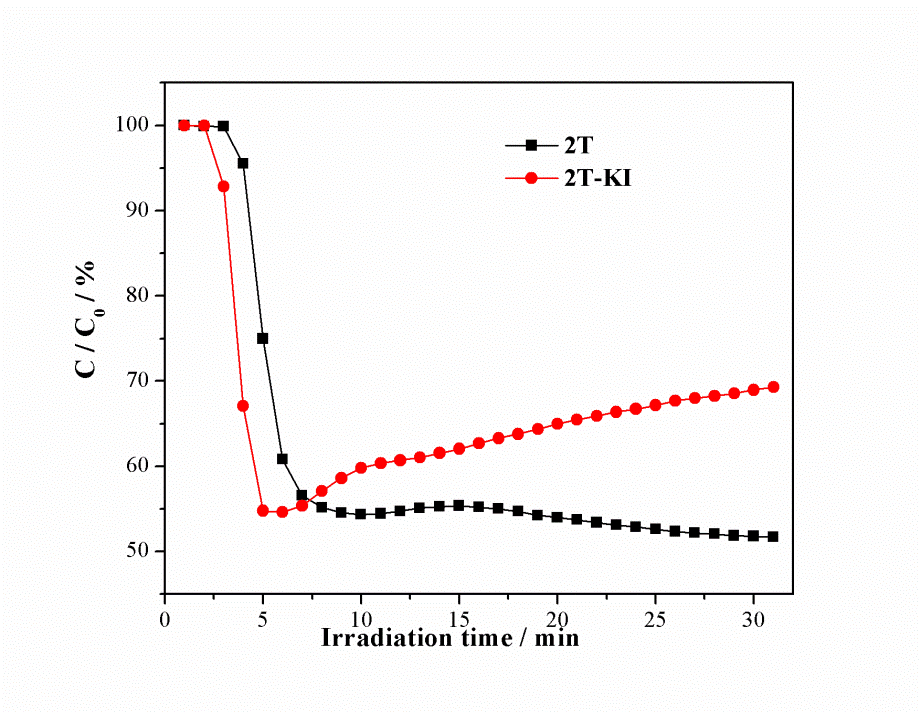


**Figure. S3** The trapping of photo-generated holes of CN-2T by KI during photocatalysis under visible light irradiation.
